# Pulmonale Mitbeteiligung bei seltenen systemischen Erkrankungen – Teil 2: Pulmonale Manifestationsformen im Rahmen lysosomaler und metabolischer Speicherkrankheiten, Phakomatosen sowie neuromuskulärer Grunderkrankungen

**DOI:** 10.1007/s00117-025-01498-y

**Published:** 2025-09-04

**Authors:** Jasmin Happe, Thomas Frauenfelder

**Affiliations:** Institut für Diagnostische und Interventionelle Radiologie, Universitätsspital Zürich, Rämistrasse 100, 8091 Zürich, Schweiz

**Keywords:** Lungenerkrankungen, Amyloidose, Lipid-Speicherkrankheiten, Diffuse pulmonale Hämorrhagie, Morbus Recklinghausen, Lung diseases, Amyloidosis, Lipid storage diseases, Diffuse pulmonary haemorrhage, von Recklinghausen disease

## Abstract

**Hintergrund:**

Pulmonale Manifestationen systemischer Erkrankungen stellen ein komplexes und diagnostisch herausforderndes Feld dar. Während die pulmonale Beteiligung bei immunologischen und hämatologischen Grunderkrankungen bereits gut beschrieben ist, rückt die Lungenbeteiligung bei seltenen genetischen und kongenitalen Systemerkrankungen – wie systemischen Speicherkrankheiten, neuromuskulären Erkrankungen und Phakomatosen – erst allmählich stärker in den Fokus der radiologischen Diagnostik.

**Ergebnisse:**

Häufig manifestiert sich die pulmonale Komponente erst sekundär, oft nach langjährigem Fortschreiten und Verschlechterung der Grunderkrankung. Die frühzeitige Erkennung pulmonaler Manifestationen ist jedoch entscheidend für das therapeutische Management und den weiteren Verlauf der Erkrankung. In der Bildgebung nimmt die hochauflösende Computertomographie (HRCT) eine Schlüsselrolle ein. Als technologische Innovation eröffnet die Photon-Counting-Detektor-CT (PCD-CT) neue diagnostische Möglichkeiten durch eine signifikant verbesserte räumliche Auflösung sowie ein optimiertes Signal-zu-Rausch-Verhältnis („signal-to-noise ratio“) im Vergleich zu einer modernen energieintegrierenden Detektor-CT (EID-CT).

**Schlussfolgerung:**

Die gezielte Anwendung dieser modernen CT-Technologien kann somit nicht nur die differenzialdiagnostische Einordnung pulmonaler Manifestationen verbessern, sondern auch eine frühzeitige therapeutische Intervention ermöglichen.

## Abstract (en)

**Background:**

Pulmonary manifestations of systemic diseases represent a complex and diagnostically challenging field. While pulmonary involvement in immunological and hematological disorders is well described, lung involvement in rare genetic and congenital systemic diseases—such as systemic storage diseases, neuromuscular disorders, and phacomatoses—is only gradually gaining increased attention in radiological diagnostics.

**Results:**

The pulmonary component often manifests secondarily, frequently after years of progression and worsening of the underlying disease. Early detection of pulmonary manifestations is crucial for therapeutic management and the overall disease outcome. In imaging, high-resolution computed tomography (HRCT) plays a key role. As a technological innovation, photon-counting detector CT (PCD-CT) offers new diagnostic opportunities by providing significantly improved spatial resolution and an optimized signal-to-noise ratio compared to state-of-the-art energy-integrating detector CT (EID-CT) systems.

**Conclusion:**

The targeted application of these advanced CT technologies can thus not only improve the differential diagnostic classification of pulmonary manifestations but also enable timely therapeutic intervention.

Die Lunge kann bei einer Vielzahl systemischer Erkrankungen in Mitleidenschaft gezogen werden (Abb. 1) – sei es direkt durch pathophysiologische Mechanismen der Grunderkrankung oder sekundär durch immunologische, vaskuläre oder metabolische Prozesse. Während pulmonale Manifestationsformen hämatologischer und immunologischer Grunderkrankungen bereits umfassend in der Literatur beschrieben sind (siehe Artikel „Teil 1“), rückt die Beteiligung der Lunge bei seltenen genetischen und kongenitalen Systemerkrankungen erst allmählich stärker in den Fokus der radiologischen Diagnostik.

**Abb. 1:**
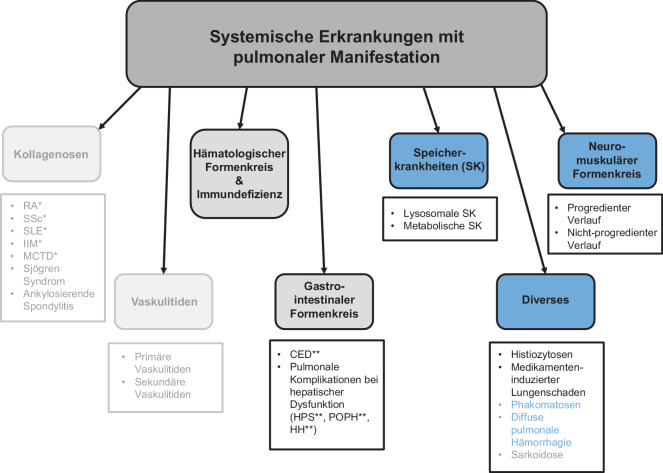
Systemische Erkrankungen mit pulmonaler Manifestation. *RA rheumatoide Arthritis, *SSc* systemische Sklerose, *SLE* systemischer Lupus erythematodes, *IIM* idiopathische inflammatorische Myositiden, *MCTD* Mischkollagenosen. **CED chronisch-entzündliche Darmerkrankungen, *HPS* hepatopulmonale Syndrome, *POPH* portopulmonale Hypertension, *HH* hepatischer Hydrothorax. Die *blau* gefärbten Bereiche werden hier, in Teil 2 dieses Artikels, behandelt. Die *grau* hinterlegten Felder sind Gegenstand von Teil 1 dieses Artikels [1]. Die *abgeblassten* Bereiche wurden bereits in drei weiteren Beiträgen dieser Ausgabe [2–4] ausführlich erläutert, gehören jedoch ebenfalls zum Formenkreis „systemischer Grunderkrankungen mit pulmonaler Manifestation“ und sind hier der Vollständigkeit halber mit aufgeführt

Gerade diese seltenen Entitäten stellen in der klinischen und bildgebenden Praxis eine besondere Herausforderung dar, da ihr systemisches Manifestationsspektrum außerordentlich breit gefächert ist. Nicht selten manifestiert sich die pulmonale Beteiligung erst sekundär – etwa im Rahmen einer primär kardialen Manifestation mit zunehmenden Zeichen einer pulmonalen Stauung – und wird oftmals erst im Verlauf klinisch und radiologisch evident. Ihre Diagnosestellung ist jedoch essenziell, da sie richtungsweisend für das weitere therapeutische Prozedere im Rahmen der Grunderkrankung sein kann.

In diesem Kontext kommt der thorakalen Bildgebung, insbesondere der hochauflösenden Computertomographie (HRCT), eine zentrale Rolle zu. Zunehmend an Bedeutung gewinnt hierbei auch die Photon-Counting-CT-Technologie, die sich im Vergleich zur herkömmlichen energieintegrierenden Detektor-CT („energy-integrating detector computed tomography“) durch eine signifikant höhere räumliche Auflösung sowie ein deutlich verbessertes Signal-zu-Rausch-Verhältnis („signal-to-noise ratio“) auszeichnet.

Auf diese Weise ermöglicht die moderne thorakale Bildgebung die frühzeitige Detektion subtiler pathologischer Veränderungen des Lungenparenchyms im Rahmen der Grunderkrankung.

Der Teil 2 dieses Beitrags widmet sich ausgewählten seltenen, überwiegend genetischen und kongenitalen Systemerkrankungen mit potenzieller pulmonaler Beteiligung, darunter systemische Speicherkrankheiten, neuromuskuläre Erkrankungen und Phakomatosen. Ergänzend wird zudem auf die pulmonale Hämorrhagie als seltene, jedoch klinisch bedeutsame Differenzialdiagnose eingegangen, die bei passender Klinik und Bildgebung stets mitbedacht werden sollte.

## Speicherkrankheiten

### Lysosomale Speicherkrankheiten

Die genetischen lysosomalen Speicherkrankheiten lassen sich grob in 3 Hauptgruppen unterteilen:

1. Lipid-Speicherkrankheiten (Morbus Gaucher, Niemann-Pick-Syndrom, Morbus Fabry),
2. Glykogen-Speicherkrankheiten (Danon-Erkrankung, Cori/Forbes-Erkrankung, PRKAG2-Mutation),
3. Hermansky-Pudlak-Syndrom [5].

**Abb. 2:**
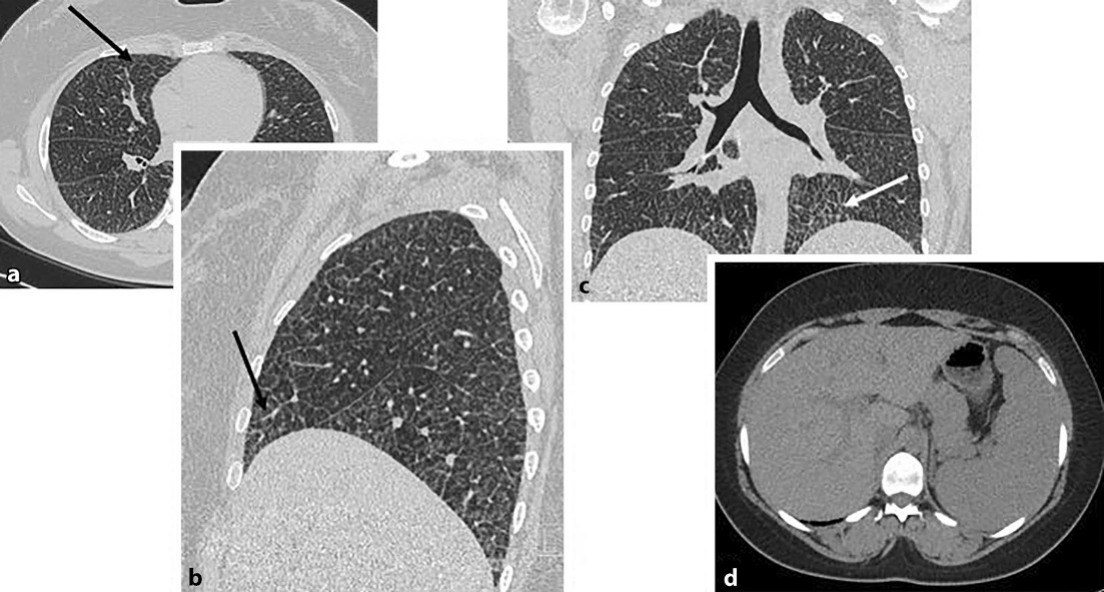
Morbus Pompe, eine seltene, erbliche Stoffwechselerkrankung, die durch einen Mangel des Enzyms saure Alpha-Glukosidase zu einer pathologischen Akkumulation von Glykogen in den Lysosomen führt. Sie äußert sich vorwiegend mit kardialer, muskuloskeletaler und hepatischer Beteiligung. Die axiale CT-Thorax auf Höhe der Lungenmittel- und Unterlappen (**a**) sowie die sagittale (**b**) und koronare (**c**) Rekonstruktion zeigen eine deutliche Verdickung der Interlobär- und Interlobulärsepten (*schwarze* und *weiße* *Pfeile*). Bildmorphologisch entsprechen diese interstitiellen Veränderungen einem Lungenödem. Dieses ist zum einen auf die häufige kardiale Beteiligung bei Morbus Pompe zurückzuführen, meist in Form einer hypertrophen Kardiomyopathie (hier nicht abgebildet). Durch die daraus resultierende diastolische Dysfunktion kommt es zu einer Druckerhöhung im linken Vorhof und einem Rückstau des Blutvolumens in die Lungenvenen. Zum anderen wird das Lungenödem durch die im Rahmen der Grunderkrankung bedingte muskuläre Schwäche der Zwerchfellmuskulatur verstärkt, die sich bildmorphologisch v. a. in einem Zwerchfellhochstand zeigt (**b** und **c**). Die axiale CT-Abdomen zeigt den Befund einer begleitenden Hepatosplenomegalie (**d**). (Mit freundl. Genehmigung, © Prof. C. Schaefer-Prokop)

Die Glykogen-Speicherkrankheiten führen in erster Linie zu einer kardialen Manifestation [5] mit sodann oft erst sekundärer pulmonaler Beteiligung im Rahmen eines kardial bedingten Lungenödems (Abb. 2).

#### Morbus Gaucher.

Unter den Lipid-Speicherkrankheiten weist der Morbus Gaucher die höchste Prävalenz auf. Die *Gaucher-Krankheit*, verursacht durch einen Mangel an Glukozerebrosidase, führt infolge der Infiltration von Gaucher-Zellen in das pulmonale Interstitium zu einer Verdickung der Interlobulärsepten [6, 7]. In einzelnen Fallberichten wurden zudem Veränderungen beschrieben, die einer desquamativen interstitiellen Pneumonie ähneln [8], mutmaßlich aufgrund einer alveolären Füllung mit Gaucher-Zellen. Das Auftreten einer pulmonalen Hypertonie wird zudem mit der Infiltration perivaskulärer, peribronchialer und septaler Regionen in Verbindung gebracht, was zu sekundär-fibrotischem Remodeling und der daraus resultierenden Okklusion kleiner alveolärer Kapillaren führt [9–11]. Die im Rahmen der Grunderkrankung auftretende Leberbeteiligung kann darüber hinaus sekundär in einem hepatopulmonalen Syndrom münden [9].

#### Niemann-Pick-Syndrom.

Das *Niemann-Pick-Syndrom* führt in verschiedenen Organen zu einer Ansammlung von Sphingomyelin [5]. In der Lunge lagern sich lipidbeladene Makrophagen, sog. Niemann-Pick-Zellen, in den Alveolarwänden, dem Interstitium und der Pleura ab [5, 6], wobei die pulmonale Architektur erhalten bleibt. Bildmorphologisch dominieren Ground-Glass-Opazitäten, führend in den Lungenoberlappen, sowie eine Verdickung der interlobulären Septen, prädominierend in den basalen Lungenregionen [6]. Histopathologisch gesicherte Case Reports deuten zudem auf eine mögliche Unterdiagnose einer diffusen endogenen Lipoidpneumonie beim Niemann-Pick-Syndrom hin [12]. Laut Nicholson et al. [12] sollte daher in ungeklärten Fällen einer diffusen Lipoidpneumonie das Niemann-Pick-Syndrom zumindest als Differenzialdiagnose in Erwägung gezogen werden.

#### Anderson-Fabry-Krankheit.

Die *Anderson-Fabry-Krankheit* (AFD; auch Morbus Fabry) betrifft primär das Herz-Kreislauf-System, wobei eine Akkumulation von Glykosphingolipiden in Myozyten zu einer (konzentrischen oder auch exzentrischen) linksventrikulären Hypertrophie und letztlich zu Myokardfibrose führen kann [5]. Eine Lungenbeteiligung kann in Form einer chronischen Atemwegobstruktion im Rahmen der pathologischen Glykosphingolipid-Akkumulation auftreten [13].

#### Hermansky-Pudlak-Syndrom.

Das *Hermansky-Pudlak-Syndrom* (HPS), charakterisiert durch eine Trias aus okulokutanem Albinismus, Thrombozytenfunktionsstörungen und pulmonaler Manifestation, präsentiert sich computertomographisch v. a. mit Retikulationen, Traktionsbronchiektasen, einer Verdickung der Interlobulärsepten sowie diffusen Ground-Glass-Opazitäten [6]. Histopathologisch zeigt sich eine fibrosierende ILD vom UIP-Pattern [14].

### Metabolische Speicherkrankheiten

Metabolische Speicherkrankheiten stellen eine heterogene Gruppe von Erkrankungen dar, zu der im systemischen Formenkreis u. a. die Amyloidose, die pulmonale Alveolarproteinose und die Hämosiderose gehören [6].

#### Pulmonale Alveolarproteinose.

Die pulmonale Alveolarproteinose (PAP) ist – wie bereits erläutert – durch eine pathologische Akkumulation von Phospholipoproteinen in den Alveolen charakterisiert und kann sekundär im Kontext verschiedener Immundefizienzsituationen oder hämatologischer Neoplasien auftreten.

#### Amyloidose.

Ein ebenfalls mit einer gestörten Proteinablagerung assoziiertes Krankheitsbild stellt die Amyloidose dar, bei der es pathophysiologisch zu einer Ablagerung abnormaler Amyloidfibrillen im extrazellulären Raum und folglich zu Gewebeschäden kommt [5]. Grundsätzlich wird zwischen *lokalen* und *systemischen* Formen der Amyloidose unterschieden: Lokale Amyloidosen sind durch die Produktion fibrillärer Proteine in einem umschriebenen Gewebe charakterisiert. Systemische Amyloidosen werden weiter unterteilt in:

1. primäre (AL‑)Amyloidose, meist basierend auf einer monoklonalen Plasmazelldyskrasie,
2. sekundäre (AA‑)Amyloidose, häufig assoziiert mit chronischen Nierenerkrankungen oder chronisch-entzündlichen Erkrankungen wie Morbus Crohn, rheumatoider Arthritis oder dem Sjögren-Syndrom,
3. familiär-hereditäre Amyloidosen, die auf genetischen Mutationen beruhen [5, 6].

**Abb. 3:**
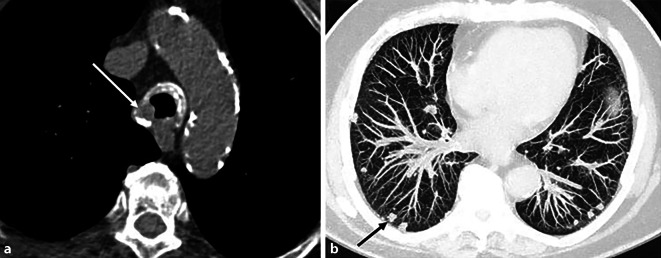
Tracheobronchiale und parenchymale Amyloidose-Manifestation. **a** CT-Thorax im Weichteilfenster eines 80-jährigen Patienten mit tracheobronchialer Manifestation einer pulmonalen Aspergillose. Bildmorphologisch zeigt sich eine zirkumferente tracheale Wandverdickung (Fortsetzung der Wandverdickung bis in die Hauptstammbronchien; hier jedoch nicht abgebildet) mit assoziierten, ausgedehnten submukosalen Kalzifikationen. Zudem plaqueartige, submukosale Trachealauflagerung rechts-laterodorsal (*weißer* *Pfeil*), vereinbar mit einer fibrillären Amyloidablagerung. **b** Maximumintensitätsprojektions(MIP)-Rekonstruktion im axialen Lungenfenster. Dargestellt ist die nodulär-parenchymale Form der pulmonalen Amyloidose bei einem anderen Patienten mit charakteristischem, überwiegend subpleuralem Verteilungsmuster der multiplen, scharf umschriebenen pulmonalen Noduli (*schwarzer Pfeil*)

**Abb. 4:**
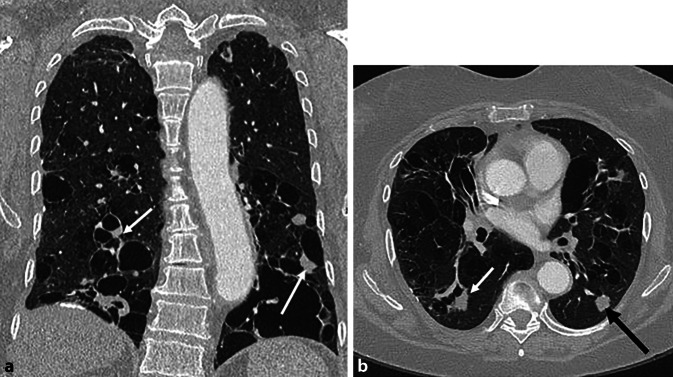
**a** Koronare Rekonstruktion. **b** Axiale CT-Thorax auf Höhe der Mittel- und Unterlappen. Äußerst seltene Manifestationsform der pulmonal-parenchymalen Amyloidose in Form einer zystischen Lungenerkrankung bei einem Patienten mit Leichtkettenamyloidose. Es sind multiple Lungenzysten mit basaler Verteilungsdominanz erkennbar sowie pathognomonische perizystische Noduli (*weiße* *Pfeile*). Darüber hinaus finden sich vereinzelt scharf umschriebene Noduli mit subpleuraler und perifissuraler Lokalisation (*schwarzer* *breiter* *Pfeil* in **b**), welche das deutlich häufiger abgrenzbare Bild der nodulär-parenchymalen Manifestationsform zeigen. (Mit freundl. Genehmigung, © Prof. C. Schaefer-Prokop)

Bildmorphologisch kann die pulmonale Amyloidose, neben einer kardialen und pleuralen Beteiligung [7] sowie einer assoziiert-begleitend auftretenden Lymphadenopathie [7], eine tracheobronchiale, parenchymal-noduläre und diffus-interstitielle Manifestationsform aufweisen [5, 6]:

- Bei der *tracheobronchialen Manifestation* kommt es zu einer submukosalen Ablagerung der pathologischen Amyloidfribrillen in Larynx, Trachea sowie Haupt- und Segmentbronchien. Dies führt zu nodulär-plaqueartigen Veränderungen oder zu einer zirkumferenten Wandverdickung der betroffenen Atemwege (Abb. 3). Sekundäre luminale Stenosen führen zur Bildung von Atelektasen oder postobstruktiven Pneumonien. Murale Kalzifikationen in den plaqueartigen Veränderungen sind charakteristisch.
- Die *nodulär-parenchymale Form* manifestiert sich in singulären oder multiplen, scharf umschriebenen, primär subpleural lokalisierten Noduli mit assoziierter Kalzifikation (Abb. 3). Kavitierende Noduli sind möglich. Zudem zeigen sich Bronchialwandverdickungen und Bronchiektasen. Charakteristisch, wenn auch selten, ist das Auftreten zufällig verteilter pulmonaler Zysten mit angrenzend perizystischen Noduli ([15]; Abb. 4).
- Die *diffus-interstitielle Form* manifestiert sich durch eine pathologische Ablagerung der Amyloidfibrillen im pulmonalen Interstitium primär in Form eines retikulären Musters, begleitet von einer Verdickung der interlobulären Septen.

#### Hämochromatose.

Ein weiteres Krankheitsbild im Formenkreis pathologischer Speicher- und Ablagerungserkrankungen stellt die *Hämochromatose* dar, die durch eine übermäßige Eisenakkumulation in verschiedenen Organen gekennzeichnet ist. Thorakal manifestiert sich diese meist in Form einer myokardialen Eisenüberladung, die bei fortschreitender Eisenablagerung strukturelle und funktionelle Veränderungen des Herzmuskels bedingen und in eine sekundäre Kardiomyopathie münden kann [16]. Darüber hinaus sind auch Eisenablagerungen in der Glandula thyroidea beschrieben [17].

Ätiologisch wird zwischen 2 Formen unterschieden:

- Die primäre (hereditäre) Hämochromatose beruht auf einem genetischen Defekt im *HFE*-Gen, der zu einer inadäquat erhöhten gastrointestinalen Eisenresorption führt [16].
- Die sekundäre Hämochromatose entsteht infolge chronischer Grunderkrankungen, die mit wiederholten Bluttransfusionen einhergehen. Typische Ursachen sind myelodysplastische Syndrome sowie hämolytische Anämien wie Thalassämie und Sichelzellanämie, die zu einer transfusionsbedingten Eisenüberladung führen [18].

Eine eigenständige Manifestationsform innerhalb eisenassoziierter Speichererkrankungen stellt die pulmonale Hämosiderose dar, die ein differenziertes Patientenkollektiv betrifft:

- Die primäre pulmonale Hämosiderose umfasst sowohl die idiopathische Form, welche typischerweise im Kindes- oder jungen Erwachsenenalter auftritt und durch rezidivierende diffuse alveoläre Hämorrhagien charakterisiert ist [19], als auch autoimmunassoziierte Formen, insbesondere im Kontext des Goodpasture-Syndroms.
- Die sekundäre pulmonale Hämosiderose entsteht typischerweise im Rahmen chronischer kardiovaskulärer Erkrankungen, v. a. bei langjähriger Mitralstenose oder kombinierten Mitralklappenvitien [6].

Bildgebend zeigt sich die pulmonale Hämosiderose durch kleine, unscharf begrenzte pulmonale Noduli sowie grobe Retikulationen mit führender Dominanz in den mittleren und basalen Lungenabschnitten [20]. Bei chronisch-persistierender Mitralstenose kann es zudem zur Ausbildung kalzifizierter oder ossifizierter, miliär verteilter Noduli kommen [20, 21].

## Neuromuskuläre Erkrankungen

Neuromuskuläre Erkrankungen (NMD) beeinträchtigen die Muskelaktivität entweder direkt durch Schädigung der Muskulatur oder indirekt durch Störungen der Nerven oder der neuromuskulären Übertragung. Sie lassen sich grob in 5 Hauptgruppen unterteilen [22]:

1. Muskuläre Dystrophien (z. B. Duchenne-Muskeldystrophie),
2. kongenitale und metabolische Myopathien (z. B. Glykogenosen),
3. Erkrankungen der neuromuskulären Übertragungsstelle (z. B. Myasthenia gravis),
4. periphere Neuropathien (z. B. Charcot-Marie-Tooth-Erkrankung),
5. Erkrankungen der Vorderhornzellen (z. B. spinale Muskelatrophien).

Im Formenkreis der NMD stellt die respiratorische Insuffizienz eine der häufigsten Todesursachen dar [22]. Sie kann entweder akut, etwa durch Pneumonien, oder chronisch durch eine progrediente ventilatorische Insuffizienz auftreten [22].

Eine fortschreitende muskuläre Dysfunktion kann hierbei die exspiratorische, inspiratorische und obere Atemwegsmuskulatur betreffen, was entsprechend zu verschiedenen Formen der Insuffizienz führt:

- Versagen der muskulären Funktion der oberen Atemwege und Beeinträchtigung der Schluckaktes,
- Versagen der Atempumpe (respiratorische Muskelinsuffizienz) und
- Versagen des Hustenreflexes [23].

Besonders die Schwäche der exspiratorischen Muskulatur, verbunden mit einer verminderten Lungen- und Thoraxelastizität, führt zu einer Reduktion des totalen Lungenvolumens (TLC) und der Vitalkapazität (VC; [22, 23]). Gleichzeitig kann es zu einem relativen Anstieg des Residualvolumens (RV) kommen, was auf eine disproportionale Schwäche der exspiratorischen gegenüber der inspiratorischen Muskulatur hinweist [22]. Die übermäßige Schwäche der exspiratorischen Muskulatur führt zudem zu einer reduzierten Effektivität des Hustens und begünstigt damit wiederum das Auftreten rezidivierender Atemwegsinfektionen [22].

**Abb. 5:**
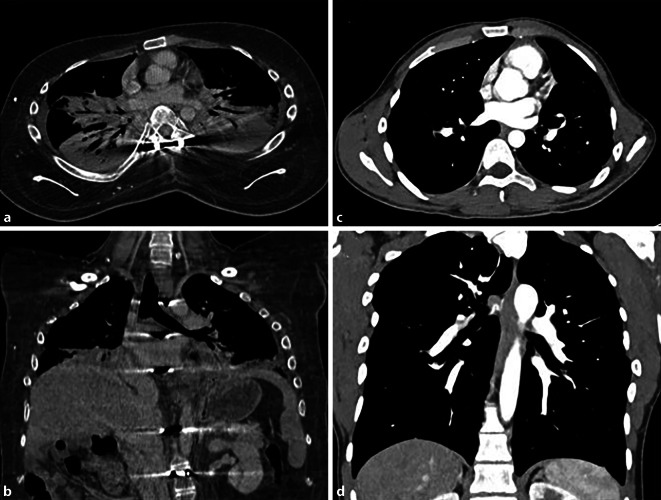
**a**, **b** CT-Thorax eines 23-jährigen Patienten mit Duchenne-Muskeldystrophie (**a** axiales Weichteilfenster, **b** koronale Rekonstruktion). **c, d** CT-Thorax eines gleichaltrigen, gesunden jungen Mannes (**c** axiales Weichteilfenster, **d** koronale Rekonstruktion). Im direkten Vergleich wird die schwere Atrophie der Skelettmuskulatur deutlich, einschliesslich der für die Atmung essenziellen Interkostalmuskulatur (**a**, **b**). Auffällig zudem die sekundäre Volumenreduktion der Lunge mit konsekutiv bilateralem Zwerchfellhochstand bei dem Patienten mit Duchenne-Muskeldystrophie (**b**). Im Vergleich dazu zeigt der gesunde Patient eine altersentsprechende Lungenkapazität mit deutlich tiefer stehenden Zwerchfellschenkeln (**d**). Zusätzlich finden sich in **a** bilaterale Konsolidationen in beiden Unterlappen, dies entspricht mutmaßlich begleitenden lobärpneumonischen Infiltraten. Diese Patienten neigen aufgrund der zunehmenden Schwäche der exspiratorischen Atemmuskulatur und der gleichzeitig reduzierten Effektivität des Hustens zu einem deutlich erhöhten Aspirationsrisiko. Rezidivierende Infektionen begünstigen im weiteren Krankheitsverlauf die Ausbildung postentzündlicher struktureller Parenchymschäden, was die Prädisposition für weitere Infektionen und somit das Risiko für Komplikationen weiter erhöht

Diese muskuläre Schwäche beeinträchtigt die Effektivität des Hustenstoßes, was wiederum die Anfälligkeit für rezidivierende Atemwegsinfektionen erhöht [7]. Bildmorphologisch zeigt sich dies in einer reduzierten Belüftung beider Lungenflügel, mit konsekutiver Atelektasenbildung und verminderten Lungenvolumina ([23]; Abb. 5). Bei gleichzeitiger oropharyngealer Inkompetenz erhöht sich das Risiko von Aspirationen und gastroösophagealem Reflux. Eine begleitend oft vorliegende Skoliose führt zu einer weiteren Beeinträchtigung der restriktiven Komponente [22].

Neben chronisch-progredienten NMD sind auch nichtprogrediente Formen wie die Zerebralparese oder statische Enzephalopathien mit relevanten pulmonalen Komplikationen assoziiert. Aufgrund von mukoidem Sekretverhalt und sekundären Bronchiektasen kann ein bronchiales Sekretmanagement mit Absaugung oder eine mechanisch assistierte Ventilation notwendig werden.

## Phakomatosen

Die Neurofibromatose Typ 1 (NF-1), auch als Morbus von Recklinghausen bekannt, ist eine autosomal-dominant vererbte Dysplasie des Ekto- und Mesoderms mit einer hohen klinischen Variabilität, weist jedoch in der Regel eine nahezu vollständige Penetranz bis zum 5. Lebensjahr auf [24]. Pulmonale Manifestationen treten hingegen typischerweise erst im Erwachsenenalter, meist in der 3. bis 4. Lebensdekade, in Erscheinung.

In der hochauflösenden CT finden sich bevorzugt:

- dünnwandige Lungenzysten,
- Bullae,
- Ground-Glass-Opazitäten,
- Noduli,
- bibasale Retikulationen,
- emphysematöse Veränderungen.

Ein charakteristischer Befund ist die gleichzeitige Präsenz einer bilateralen, basalen retikulären Zeichnungsvermehrung bei gleichzeitigen Bullae und Zysten in den apikalen Lungenabschnitten [24]. Ein eindeutiger Zusammenhang zwischen NF‑1 und dem Auftreten interstitieller Parenchymalterationen bleibt allerdings weiterhin Gegenstand wissenschaftlicher Diskussion, da bislang nur unzureichende Evidenz für eine direkte Assoziation vorliegt [25].

**Abb. 6:**
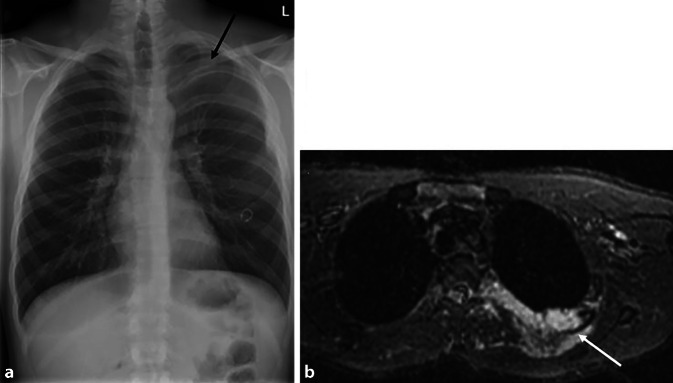
Morbus Recklinghausen. Die p. a.-Röntgenaufnahme des Thorax (**a**) zeigt eine ausgeprägte Densitätsasymmetrie der Lungenoberlappen mit flächiger Transparenzminderung links apikal (*schwarzer Pfeil*). In der ergänzenden MRT (**b**) zeigt sich eine STIR-hyperintense Läsion interkostal links dorsal (*weisser* *Pfeil*) mit ausgedehntem umgebendem Weichteilödem – ein charakteristisches Bild für ein Neurofibrom im Rahmen eines Neurofibromatose-Typ-1-Syndroms (NF1)

Weitere bildmorphologische Merkmale umfassen:

- kutane und subkutane Neurofibrome der Thoraxwand (Abb. 6),
- mediastinale Raumforderungen (z. B. plexiforme Neurofibrome, Meningozelen),
- sowie muskuloskeletale Veränderungen, darunter:
  - Kyphoskoliose,
  - posteriore Scallopings der Wirbelkörper,
  - Erweiterung der Neuroforamina,
  - Rippendeformitäten infolge ossärer Dysplasien oder Erosion durch benachbarte Neurofibrome [24].

In selteneren Fällen kann es auch zur Manifestation einer pulmonalen Hypertonie kommen.

Zur Gruppe der Phakomatosen werden darüber hinaus die tuberöse Sklerose (TS) sowie die Lymphangioleiomyomatose (LAM) gezählt. Es wird vermutet, dass die LAM eine unvollständige phänotypische Ausprägung der tuberösen Sklerose darstellt.

**Abb. 7:**
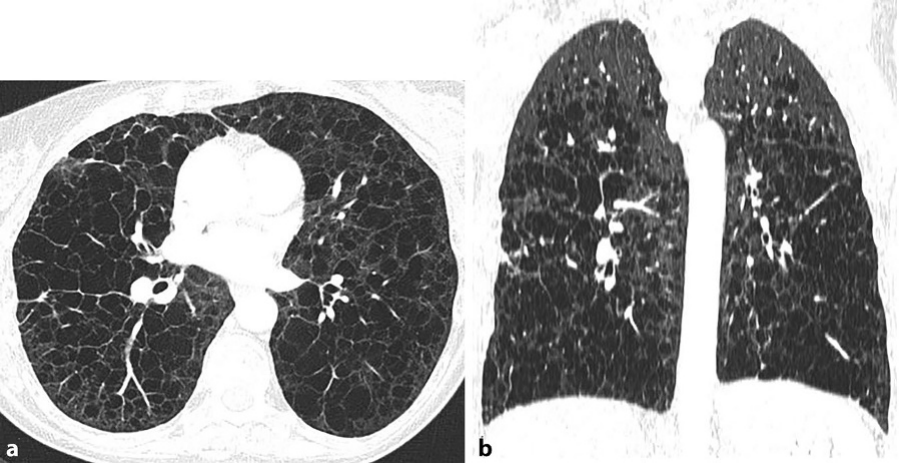
CT einer 58-jährigen Patientin mit bekannter Lymphangioleiomyomatose (LAM) zur Abklärung einer möglichen Lungentransplantation. **a** Axiales Lungenfenster, **b** koronale Rekonstruktion im Lungenfenster. Bild einer ausgedehnten zystischen Lungenerkrankung mit nur noch wenig residuell verbleibendem Lungenparenchym, insbesondere in den Mittel- und Unterlappen führend. Die Lungenzysten zeigen im Vergleich zur Langerhans-Zell-Histiozytose (LZH; s. Abb. 4 in „Teil 1“) eine dünnwandige, rundlich-homogene Kontur, während die Zystenkonfiguration bei der LZH unregelmäßig und bizarr erscheint. Das Bild könnte leicht mit einem Lungenemphysem verwechselt werden, wobei eine fehlende – für das Emphysem jedoch typische – apikale Prädominanz hier differenzialdiagnostisch hilfreich sein kann. Bei der Patientin sind keine Angiomyolipome bekannt

Bildmorphologisch zeigt sich ein dominierendes Muster einer zystischen Lungenerkrankung, welches durch ubiquitäre, dünnwandige, runde Lungenzysten gekennzeichnet ist, die keine spezifische kraniokaudale oder axiale Verteilungspräferenz aufweisen [26]. Ohne therapeutische Intervention kommt es zu einer fortschreitenden Zunahme der Anzahl und Größe der Lungenzysten, sodass schließlich nur noch wenig normales Lungengewebe verbleibt (Abb. 7). In fortgeschrittenen Stadien kann dies fälschlicherweise mit einem Lungenemphysem verwechselt werden. Pathologisch liegen inaktivierende Mutationen der TSC 2- oder TSC 1-Tumorsuppressorgene vor, die zu einer pathologischen Proliferation von LAM-Zellen führen. Diese infiltrieren Lymphgefäße, Atemwege und Blutgefäße, was zu Obstruktionen und Rupturen führt. Dies wiederum begünstigt:

- pulmonale Hämorrhagien,
- chylöse Pleura- oder Perikardergüsse sowie
- spontane, häufig rezidivierende Pneumothoraces infolge von Zystenrupturen.

Zusätzlich können sich kleine Noduli in Form einer multifokalen mikronodulären Pneumozytenhyperplasie (MMPH) zeigen – eine typische Manifestation der tuberösen Sklerose.

Im Krankheitsverlauf kann es zu einer respiratorischen Insuffizienz infolge des fortschreitenden Verlusts funktionellen Lungengewebes kommen [26].

## Diffuse pulmonale Hämorrhagie

Die pulmonale Hämorrhagie, insbesondere in Form der diffusen alveolären Hämorrhagie (DAH), stellt eine seltene, aber differenzialdiagnostisch relevante Entität bei akut auftretenden alveolären Verschattungen im Rahmen systemischer Grunderkrankungen dar. Histologisch kann die DAH als Folge einer pulmonalen Kapillaritis auftreten – einer nekrotisierenden Vaskulitis mit Kapillarwandnekrosen, die häufig mit Immunkomplexablagerungen assoziiert ist [7]. Ein klassisches Beispiel hierfür stellt das Goodpasture-Syndrom dar, bei dem typischerweise eine DAH gemeinsam mit einer rasch progredienten Glomerulonephritis auftritt. Allerdings kann eine diffuse alveoläre Hämorrhagie auch bei zahlreichen anderen Erkrankungen auftreten, ohne dass histologisch eine Kapillaritis nachweisbar ist [7].

Radiologisch manifestiert sich die plötzliche Einblutung in die Alveolen – je nach Ausmaß – in der CT als diffuse Ground-Glass-Opazitäten bis hin zu konsolidierenden Arealen [27]. Charakteristischerweise besteht ein zentral betontes, perihiläres und basales Verteilungsmuster, wobei die Lungenperipherie, die Apices sowie die kostophrenischen Winkel typischerweise ausgespart bleiben. In den Tagen nach einem akuten Blutungsereignis kann es zur Verdickung der interlobulären Septen bei gleichzeitiger Persistenz der Ground-Glass-Opazitäten kommen – ein bildmorphologisches Muster, das als „crazy paving“ bezeichnet wird [19]. Wiederholte hämorrhagische Episoden können langfristig zu Fibrosierung des interstitiellen Gewebes, einer Verdickung der alveolären Basalmembran sowie zur Ausbildung einer pulmonalen Hämosiderose führen. Diese Veränderungen können in eine sekundäre pulmonale Hypertonie münden [7].

**Abb. 8:**
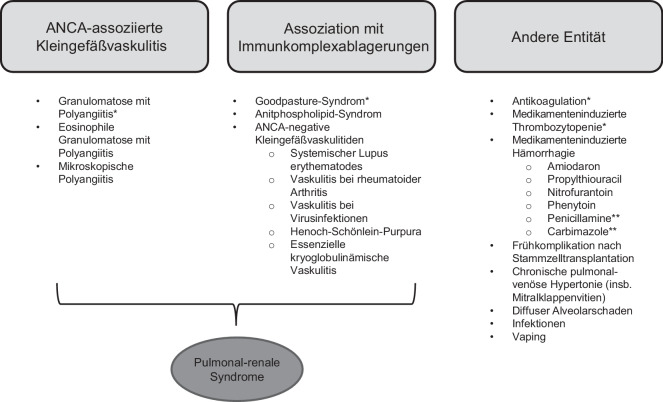
Ursachen diffuser pulmonaler Hämorrhagie (DPH). Die DPH tritt meist sekundär im Rahmen einer Grunderkrankung auf, am häufigsten bei Granulomatose mit Polyangiitis, gefolgt vom Goodpasture-Syndrom. Ätiologisch lassen sich die Ursachen grob in Vaskulitiden, Autoimmunerkrankungen sowie medikamenteninduzierte Formen unterteilen. Idiopathische Verläufe sind selten. Die pulmonal-renalen Syndrome umfassen sowohl die ANCA(antineutrophile zytoplasmatische Antikörper)-assoziierten Kleingefäßvaskulitiden als auch Erkrankungen, die mit Immunkomplexablagerungen einhergehen. *Häufigste Ursache der DPH in der jeweiligen Kategorie. **Penicillamine und Carbimalzole können in seltenen Fällen zu einem pulmonal-renalen Syndrom, ähnlich dem Goodpasture-Syndrom, führen

Differenzialdiagnostisch ist die Abgrenzung zum Lungenödem oder einer diffusen Pneumonie oft schwierig [28]. Ein mögliches Unterscheidungsmerkmal zum kardialen Lungenödem bietet die zeitliche Dynamik: Während die Ground-Glass-Opazitäten bei der DAH typischerweise über einen Zeitraum von mehreren Tagen bis hin zu 2 Wochen resorbiert werden, so erfolgt die Rückbildung beim kardialen Lungenödem meist deutlich rascher. Generell sollte eine DAH differenzialdiagnostisch bei Vorliegen (bereits milder) Hämoptysen oder bei Vorliegen DAH-assoziierter Ursachen in Erwägung gezogen werden (eine Übersicht möglicher Ursachen der DPH bietet Abb. 8).

## Praxistipps – Teil 2

- Ein hilfreiches Unterscheidungsmerkmal zur Differenzierung zwischen einem kardialen Lungenödem und der diffusen pulmonalen Hämorrhagie (DPH) ist die zeitliche Dynamik: Während sich die pulmonalen Veränderungen bei DPH über Tage bis 2 Wochen zurückbilden, erfolgt dies beim kardialen Lungenödem deutlich schneller.
- Bei diffusem Ground-Glass-Muster sollte eine pulmonale Hämorrhagie – v. a. im Kontext systemischer Grunderkrankungen – als Differenzialdiagnose erwogen und in enger Abstimmung mit der Klinik abgeklärt werden.
- Bei systemischen Erkrankungen mit diffusen parenchymatösen Lungenveränderungen ist es essenziell, das dominierende Muster zu identifizieren. Dieses liefert häufig den entscheidenden Hinweis für die zugrundeliegende Pathologie und verhindert diagnostische Fehlinterpretationen.

## Fazit für die Praxis

- Insgesamt liefert die Identifikation und präzise diagnostische Einordnung charakteristischer pulmonal-radiologischer Muster bei systemischen Grunderkrankungen wesentliche Hinweise auf die zugrundeliegende Pathologie und unterstützt auf diese Weise die klinisch-laborchemische Korrelation im Rahmen eines integrativen diagnostischen Ansatzes.
- Die Bildgebung ermöglicht somit die frühzeitige Erkennung subtiler Veränderungen des Lungenparenchyms und trägt entscheidend zur differenzialdiagnostischen Einordnung seltener pulmonaler Manifestationsformen bei.
- Aufgrund der diagnostischen Komplexität ist eine enge interdisziplinäre Zusammenarbeit zwischen Klinikern, Radiologen und Pathologen unerlässlich, um Diagnosen frühzeitig zu sichern, Therapieentscheidungen zu optimieren und die Patientenversorgung nachhaltig zu verbessern.
